# Natural polymers in diabetes management: formulation prospects of *Hibiscus rosa-sinensis* mucilage for metformin sustained release

**DOI:** 10.3389/fbioe.2025.1735314

**Published:** 2026-01-12

**Authors:** Nsibambi Ronald Kizza, Sarad Pawar Naik Bukke, Ungo-Kore Hussain Yahaya, Yakubu Sunday Bot

**Affiliations:** 1 Department of Pharmaceutics and Pharmaceutical Technology, Kampala International University, Ishaka, Bushenyi, Uganda; 2 Department of Medical Laboratory Science, Kampala International University, Ishaka, Bushenyi, Uganda

**Keywords:** diabetes management, *Hibiscus rosa-sinensis* mucilage, matrix tablets, metformin, natural polymers, sustained release

## Abstract

Diabetes mellitus (DM) is a long-term metabolic disorder distinguished by hyperglycemia with the limitation of severe complications unless treated effectively. Metformin is the first-line agent for treating diabetes type 2 but has the drawback of a short half-life, incomplete absorption and is associated with gastrointestinal side effects requiring multiple times a day dosing. Sustained release (SR) formulations have been explored for the delivery of metformin while prolonging plasma levels, decreasing the frequency of dosing and increasing adherence improve patient compliance and outcomes related to diabetes. Some natural polymers have attracted attention as excipients for SR systems because they are biocompatible, biodegradable, and cost-effective. Specifically, *Hibiscus rosa-sinensis* mucilage has shown potential to be clinically useful due to its functional properties such as a high swelling index, gel-forming ability, stabilization of a matrix and compatibility with synthetic polymers. The extraction of the mucilage from the leaves and flowers is convenient, environmentally friendly and produces a stable hydrophilic pH stable and non-toxic polymer suitable for pharmaceutical applications. The use of a mucilage matrix in tablet formulations as well as hydrogels, beads, and/or nanoparticle systems would facilitate controlled diffusion and/or erosion -based metformin release designed to achieve zero order or Higuchi release kinetics. Additionally, the polymer would have the added therapeutic benefit due to its antioxidant and antidiabetic properties. Challenges of using mucilage include batch variability, microbial stability and limited clinical/non-clinical scientific data that indicated the need for the development of standardization and evaluation of its use. Future work includes the development of polymer.

## 1 Introduction

Diabetes mellitus (DM) represents one of the most common and fastest-growing metabolic conditions worldwide, with chronic hyperglycemia due to errors in insulin secretion, action, or both (Kumar and Kumar, 2020). The International Diabetes Federation (IDF) estimates that globally in adults, the prevalence of diabetes will increase from 537 million in 2021 to over 643 million in 2030, largely due to cases in India and other developing nations (Ogurtsova et al., 2017). Sustained hyperglycemia in individuals with diabetes leads to long-term complications, including cardiovascular disease, nephropathy, neuropathy, retinopathy, and delayed wound healing. These complications diminish quality of life and come with serious socio-economic consequences, which emphasizes the need for efficacious approaches to therapy that are convenient and patient-centered (Guariguata et al., 2011).

**FIGURE 1:**
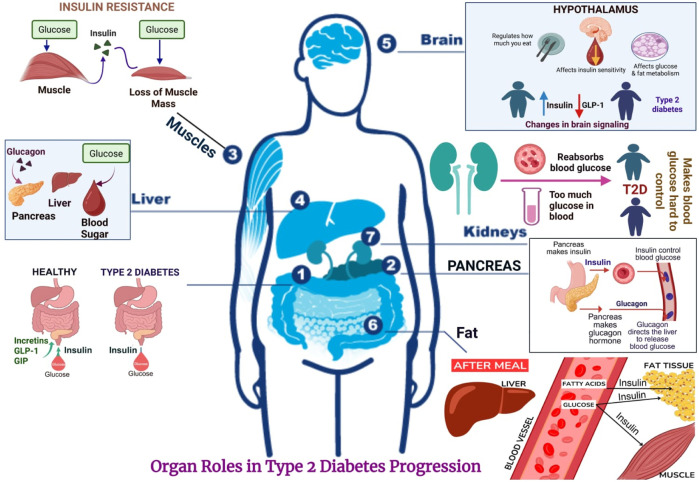
Overview of organ crosstalk in type 2 diabetes (or) pathophysiological mechanisms and organ involvement in type 2 diabetes mellitus.

Figure 1, schematic representation showing organ-specific roles in the progression of Type 2 Diabetes Mellitus, including pancreatic β-cell dysfunction, hepatic insulin resistance, adipose tissue inflammation, and impaired muscle.

Metformin, a derivative of biguanide, is the recommended gold standard first-line oral hypoglycemic agent for use in type 2 diabetes mellitus (T2DM) (Sirto et al., 2024). Its primary mechanisms of action are decreasing hepatic glucose production, increasing insulin sensitivity, and increasing peripheral glucose uptake. Despite having established being effective, well tolerated, safe, and inexpensive, metformin therapy is limited by its half-life of approximately 4–6 h (Bailey, 2024). As a result, patients must take multiple doses each day to maintain effective drug concentrations in circulation. Multiple doses daily decreases patient compliance and may complicate daily blood glucose variable (Jia et al., 2019).

In order to address these drawbacks, sustained-release (SR) formulations of metformin have been developed (Karna et al., 2015). Sustained-release drug delivery systems can provide a number of advantages, such as prolonged maintenance of plasma drug concentrations within the therapeutic window, decreased dosing frequency, improved patient adherence, and less gastrointestinal side effects, compared to immediate-release formulations (Gupta, 2012). In addition, SR formulations aid in achieving steady-state pharmacokinetics and can improve overall efficacy of treatment for chronic diseases like diabetes that require lifelong treatment.

Recently, natural polymers have received significant interest as an alternative excipient for sustained-release formulations (Wu and Jin, 2008). As renewable resources, these polymers are generally safe, biocompatible, biodegradable, and cheaper than synthetic polymers. Moreover, natural polymers have the ability to swell, gel, or produce viscous dispersions, making them well-suited candidates for drug release via diffusion or erosion. Natural polymers are also less likely to give chronic patients toxicity or adverse reactions, which is important for chronic disease management (Debotton and Dahan, 2017). Out of all the various natural polymers available, plant-derived mucilages are versatile excipients due to their abundance, eco-friendliness, and functional properties (Heer, 2013).

*Hibiscus rosa-sinensis*, which is also known as the Chinese hibiscus, has been identified as a good source of mucilage with suitable pharmaceutical properties such as high swelling index, non-toxicity and good film-forming ability (Ross, 2003). Mucilage isolated from the leaves and flowers has a real potential as a natural polymer for playing the role of a sustained release matrix for drug delivery. The application of non-toxic mucilage from *Hibiscus rosa-sinensis* as a polymeric excipient for sustained release metformin formulations could provide an innovative solution at cost effective and in a patient-friendly manner for the management of diabetes (Anderson, 2006).

The aim of this review is to present considerations for available formulations based on *Hibiscus rosa-sinensis* mucilage for sustained release metformin, examining the overall performance and role of natural polymeric excipients to enhance therapeutics in diabetes mellitus.

## 2 Natural polymers in drug delivery and diabetes management

The application of polymers in drug delivery systems has changed the landscape of contemporary pharmaceutical research and allowed for the creation of formulations capable of controlled drug release, improving drug bioavailability, and enhancing therapeutic effectiveness (Ghazwani et al., 2025). Among these polymers, natural polymers are of particular importance due to their intrinsic properties of biodegradability, biocompatibility, nontoxicity, renewability and cost effectiveness over synthetic polymers (Qiu et al., 2022). Natural polymers derived from plant, animal, or microbial sources have structural variations and functional groups to enhance their versatility in drug delivery, making them well-suited for use in developing novel drug delivery systems (Sonia and Sharma, 2012).

### 2.1 General role of natural polymers in drug delivery

Natural polymers may also serve multiple roles in drug delivery as binders, disintegrants, gelling agents, stabilizers, coating materials, and matrix formers in various formulations (Sharma et al., 2019). Furthermore, their physicochemical properties (swelling, gel formation, viscosity and films) are also exceptionally advantageous in the design of sustained release (SR) systems. For oral dosage forms, natural polymers can modulate drug release through mechanisms like diffusion, erosion, or swelling-controlled transport, thereby maintaining steady plasma drug concentrations (Ngwuluka et al., 2014). In addition, their ability to mimic physiological environments enhances the wellbeing and tolerability of formulations. This review deals the some of widely studied natural polymers include: Plant-derived polysaccharides such as guar gum, xanthan gum, gum acacia, pectin, and mucilages from various plants (okra, fenugreek, plantago, hibiscus). Marine polysaccharides like alginate and carrageenan, used for gel-forming and mucoadhesive applications (Idrees et al., 2020). Animal-derived polymers such as chitosan (from chitin) and gelatin, which exhibit excellent biocompatibility and mucoadhesive properties. Microbial polysaccharides such as pullulan and dextran, offering unique viscosity and film-forming properties. These polymers not only ensure controlled release but also provide stability against harsh gastrointestinal (GI) conditions, making them highly suitable for chronic conditions requiring long-term therapy (Sung and Kim, 2020).

### 2.2 Natural polymers in diabetes management

Natural polymers have gained prominence in modern pharmaceutics due to their ability to act as functional excipients in advanced drug delivery systems. A major function of natural polymers is in the design of controlled and extended-release systems, wherein the drug is released over an extended period of time in a controlled manner, providing a prolonged therapeutic effect while reducing the need for frequent dosing and administration (Zhao et al., 2023). This can be particularly advantageous for patients with chronic illnesses, such as diabetes mellitus, which requires adherence to a long-term treatment plan. Another important function of natural polymers is in mucoadhesion, or the ability of dosage forms to adhere to mucosal surfaces (e.g., those within the gastrointestinal tract) (Zhou et al., 2024). Developing mucoadhesive systems based on natural polymers may provide benefits for drugs with limited absorption windows or those that have short half-lives (Sonia and Sharma, 2012).

Natural polymers are also known for their relatively compatible and non-toxic properties, as they come from renewable sources found in nature, such as plants, algae, and even microbes. As natural materials, they are biodegradable, can accommodate long-term use, and can have lower risks of inducing adverse immune reactions compared to some synthetic excipients (Debotton and Dahan, 2017). At the same time, the natural source and preparation of dosing may take on even greater importance with the increasing push towards sustainability and green practice within pharmaceutical/design development.

### 2.3 Commonly used natural polymers

Various natural polymers have been investigated as excipients for controlled drug delivery systems, particularly for diabetes management. Examples include: Guar gum - a galactomannan polysaccharide that swells and is viscous, which acts as a matrix former for sustained release tablets. Xanthan gum - a microbial polysaccharide that provides thickening and gel-forming properties for many controlled release formulations (Kulkarni et al., 2012). Pectin - a plant-based polysaccharide that gels, for use in colon-targeted as well as sustained release formulations. Locust bean gum - a galactomannan that exhibits a synergistic gelling property in the presence of other gums, well-suited for oral delivery (Qiu et al., 2022). Tragacanth gum - a natural gum with superior water-binding capacity, often used for suspending and controlling release. Starch derivatives - modified starches that have good film-forming and matrix-forming possibilities are flexible excipients (Ahmed and Thornalley, 2007). The benefits of these polymers is that they are renewable, regulatory acceptance as a GRAS (Generally Recognized as Safe) substance, and exhibit structural diversity for functional modifications; however, they also have challenges that they are often inherent due to employer variability in batches that exist due to environmental and geographical conditions. These natural polymers can be impacted by contaminating microorganisms during storage which could potentially restrict their use as safe excipients. Assessment of the functionality of natural polymer excipients for good pharmaceutical quality should also consider the appropriate levels of materials to standardize drug delivery action for their reproducibility.

### 2.4 Relevance in diabetes therapy

Management of diabetes mellitus is dependent on constant and vigilant monitoring of blood glucose levels to minimize both acute and long-term complications from the disease (Ahmed and Thornalley, 2007). An example of this is with conventional immediate-release dosage forms of the drug metformin that often result in variability in drug plasma concentration resulting in peaks and doses that might risk gastrointestinal irritation. Natural polymers used in sustained release systems can minimize fluctuations and allow for a more consistent rate of drug release and more stable glycemic control (Huang et al., 2018).

In addition to their function as inert excipients, some natural polymers can themselves have synergistic or therapeutic effects in the management of diabetes. Guar gum and fenugreek mucilage, for example, have been reported to delay glucose absorption, decrease postprandial glucose spikes, and improve lipid profile (Bradley and Speight, 2002). Natural polymers also frequently exhibit antioxidant or hypoglycemic activities that can act in addition to the pharmacological effects of antidiabetic drugs. This dual effect gives them unique advantages in the development of novel and appropriate drug delivery systems to improve therapy in the management of diabetes (Currie et al., 2013).

In this regard, *Hibiscus rosa-sinensis* mucilage is a particularly attractive natural polymer due to its excellent swelling index, film forming ability, and general riskfree, all of which make it an appropriate natural polymer for sustained release formulations with metformin.

## 3 *Hibiscus rosa-sinensis* mucilage: source and properties

### 3.1 Botanical overview

*Hibiscus rosa-sinensis*, referred to as the Chinese hibiscus, shoe flower, or China rose, is a perennial evergreen shrub that is part of the Malvaceae family. This plant is native to East Asia; however, it is cultivated as an ornamental plant throughout many tropical and subtropical areas of the world due to its large and colorful flowers (Vignesh and Nair, 2018). Apart from its aesthetic uses, this plant has long been established in traditional medicine systems (e.g., Ayurveda, Unani, Chinese medicine) to treat a variety of conditions. *Hibiscus rosa-sinensis* has green, shiny leaves, trumpet-shaped flowers in all colors (most commonly red), and tissues rich in mucilage (primarily the leaves and petals) (Sivaraman and Saju, 2021). Mucilage from *Hibiscus rosa-sinensis* is a polysaccharide complex that has demonstrated remarkable swelling, viscosity-inducing, and gel forming abilities, making it useful as a pharmaceutical excipient. In traditional medicine, different parts of *Hibiscus rosa-sinensis* have been used in folk medicine to treat a variety of conditions, including, wound healing - The leaves and flowers have been applied topically as a demulcent to aid in the healing of cuts, burns, and ulcers (Bala et al., 2022). Anti-inflammatory activity–Extracts of the plant are reported to reduce inflammation and pain, supporting its use in conditions such as arthritis and inflammatory skin disorders (Gunathilaka and Geeganage, 2024). Antidiabetic effects–Several studies have demonstrated the ability of *Hibiscus rosa-sinensis* extracts to lower blood glucose levels. Mechanisms proposed include inhibition of carbohydrate-digesting enzymes (α-amylase, α-glucosidase), improvement in insulin sensitivity, and enhancement of peripheral glucose uptake (Selvan and Ramesh, 2024). This antidiabetic potential aligns with the therapeutic goals of modern diabetes management. Other ethnomedicinal uses–The plant has also been traditionally used as an emollient, hair tonic, anti-fertility agent, and in the management of hypertension and hyperlipidemia.

*Hibiscus rosa-sinensis* is a valuable mucilage source from a pharmaceutical standpoint thanks to its plentiful supply, ease of preparation, and eco-friendliness (Bala et al., 2022). Essentially, the mucilage’s functional characteristics (swelling, capacity for water retention, binding action, and film-forming ability) lend themselves to usefulness for controlled and sustained release applications. Moreover, with the plant’s recognized antidiabetic potency in traditional medicine, they provide a strong rationale for examining if the mucilage can be used as an alternative natural excipient for sustained release delivery of metformin and other antidiabetic medications (Pillai and Saraswathy, 2016).

### 3.2 Extraction and isolation

The mucilage from *Hibiscus rosa-sinensis* may be reliably sourced from the plant’s leaves and flowers since they have a significant amount of water-soluble polysaccharides (Ruban and Gajalakshmi, 2012). The extraction typically involves using water to extract the mucilage from the relevant plant parts, followed by the precipitation of the mucilage with organic solvents to isolate it from other constituents of the plant (e.g., chlorophyll, flavonoids, and secondary metabolites).

#### 3.2.1 General extraction process

Collection and Preparation of Plant Material: Fresh leaves or flowers are collected, washed thoroughly to get rid of dust and trash, and shade-dried indoors to protect any thermolabile fractions. The material is then ground to produce a coarse powder (Gunathilaka and Geeganage, 2024).

##### 3.2.1.1 Aqueous extraction

The powdered plant material is soaked or boiled in distilled water for several hours. Heat and agitation will help release the mucilage into the aqueous phase. The slurry is usually filtered through muslin cloth to eliminate the insoluble fraction.

##### 3.2.1.2 Concentration

The aqueous extract would then be concentrated using reduced pressure, or by a gentle drying process to reduce the samples volume.

##### 3.2.1.3 Precipitation

The concentrated extract is then mixed with an excess of an organic solvent such as acetone or ethanol. As the mucilage is predominantly hydrophilic, it precipitates out of the system, whilst the related impurities remain soluble in the solvent system.

##### 3.2.1.4 Purification and drying

The precipitated mucilage is filtered off from the supernatant, and solvent-washed to remove the residual impurities, and thermally dried at controlled tolerable temperatures. The dried material is ground to powder and stored in airtight containers for further use.

This is a simple, economical and environmentally friendly extraction procedure which can be used for large-scale production of nature derived excipients.

#### 3.2.2 Physicochemical properties of Hibiscus mucilage

Mucilage from the *Hibiscus rosa-sinensis* (HRS) leaf has both physical (flowability, compressibility) and chemical (structure, molecular weight and intrinsic viscosity) properties that are suitable for fabricating hydrophilic matrix polymeric (HMP) materials. The molecular weight (Mw) of HRS mucilage is ∼10 million daltons with an intrinsic viscosity of ∼23.2 in solution. Thus, HRS mucilage produces high Mw polymers that are likely to form viscous hydrogels upon hydration (Fadila et al., 2025). HRS mucilage has a backbone polysaccharide structure with a relatively high content of several sugar units (L-Rhamnose, D-Galactose, D-Galacturonic Acid and D-Glucuronic Acid), with a molar ratio of approximately 5:8:3:2. This provides for a significantly high degree of water-binding (Fadila et al., 2025).

The physical characteristics of dry, powdered HRS mucilage provide desirable properties for direct compression tablet manufacture; in powder form it has the ability to flow well and is highly compressible (angle of repose ∼25°, carr index 9%–10% and hausner ratio ∼1.1) (Lai et al., 2024).

Swelling studies indicate that HRS mucilage swells profusely in aqueous Solution; it has a swelling ratio of ∼9 in water, ∼10 in simulated gastric fluid (0.1N Hcl) and ∼9 in phosphate buffer (pH 6.8), indicating it has pH-independent swelling characteristics (Kaur et al., 2025).

The fact that HRS mucilage exhibits pH-independent swelling behaviour is advantageous for developing sustained release formulations using a hydrophilic Matrix type system, as it allows for the matrices to behave similarly by absorbing moisture and swelling during both gastric and intestinal dissolution (Singh et al., 2024).

Experimental formulations using HRS mucilage as a matrix polymer have shown sustained drug release over periods of 12–24 h with different drugs (Ketoprofen, Gabapentin, or Diclofenac) using a variety of formulations (Rocha et al., 2022).

The diffusion and erosion mechanisms of release kinetics are typically found to be non-Fickian. The results indicate that HRS mucilage has potential as a biocompatible matrix polymer for drug delivery for many years.

Metformin is highly water-soluble and, therefore, the use of HRS mucilage could provide a potential way to slow the release of Metformin from the matrix. There is now sufficient physicochemical and formulation information available to allow one to design an optimal sustained release matrix, e.g., with approximately 30%–50% w/w concentration of polymer and/or direct compression or wet granulation methods. The reduction in the amount of Metformin that is able to be released during a specified time, such as 12–24 h, is a goal of this investigation (Rodrigues et al., 2023).

**FIGURE 2:**
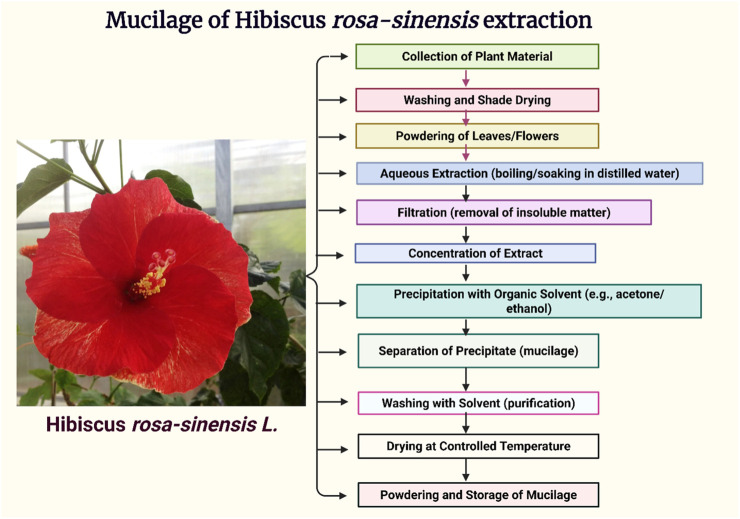
Schematic representation of the extraction process of *Hibiscus rosa-sinensis* mucilage.

The Figure 2, Illustrates the sequential steps involved in isolating mucilage from *Hibiscus rosa-sinensis* leaves, including collection, drying, powdering, extraction, filtration, and precipitation, followed by drying and storage of the purified mucilage.

### 3.3 Functional characteristics for formulation

The mucilage derived from *Hibiscus rosa-sinensis* possesses several functional properties that make it excellent as a natural excipient for drug delivery systems, especially in extended-release formulations. These properties stem primarily from its polysaccharide-rich composition, which imparts unique physicochemical and biopharmaceutical advantages (Saidin et al., 2022).

#### 3.3.1 High swelling index and water retention

Hibiscus mucilage demonstrates a pronounced ability to absorb water and swell, forming hydrated matrices upon contact with aqueous environments. The high swelling index enables slow penetration of gastrointestinal fluids into the dosage form, and hence controls drug release by controlling the diffusion pathway length (Vignesh and Nair, 2018).

In extended-release formulations, this controlled hydration diminishes or eliminates the burst release phenomenon, and prolongs drug availability over an extended period of time. For metformin which is highly soluble in water and dissolves rapidly, the swelling characteristic of Hibiscus mucilage provides a slower uniform release leading to more consistent plasma drug levels (Tomoda et al., 1987).

#### 3.3.2 Gelling and thickening capacity

The mucilage will disperse into water to form viscous gels which demonstrate the excellent thickening efficacy and the ability to modulate rheology and transform from a heterogenous solution into a gel. Gels have the ability to provide diffusion barriers which regulate the transport of drug across the hydrated matrix. This gel forming property of mucilage is especially useful in formulations that are hydrophilic matrix tablets, since drug release is governed by both swelling of the polymer and viscosity of the gel. In addition, the thickening of mucilage improves stability of formulations to improve sedimentation of drug in liquid dosage forms, and increases uniformity of drug distribution in solid dosage forms (Kharwade et al., 2017; Chowdhury et al., 2017; Ash et al., 2019).

#### 3.3.3 Ability to form stable matrices for sustained drug release

One of the key functional characteristics of Hibiscus mucilage is its ability to create stable drug-polymer matrices. Once compressed into a tablet, the mucilage adds mechanical strength and structural stability, delaying the onset of disintegration in the gastrointestinal tract (Wadher et al., 2011). The matrices are designed to erode and swell in a controlled manner, allowing extended drug release. Due to its film-forming property, the mucilage can also play a role in coating technologies for additional drug release modification. Matrix-forming characteristics are especially beneficial for drugs such as metformin since prolonged release improves the therapeutic effect and frequency of dosing (Dalvadi et al., 2013).

#### 3.3.4 Biodegradable and compatibility with synthetic polymers

As a natural polysaccharide, Hibiscus mucilage is biodegradable, allowing it to exit the body in a safe manner without toxic residues. Moreover, its natural origins provide acceptable biocompatibility for use in chronic conditions such as diabetes (Shiam et al., 2025). Hibiscus mucilage also has demonstrated strong compatibility with synthetic polymers (hydroxypropyl methylcellulose, carbopol, polyethylene oxide). This compatibility allows it to be incorporated into polymer blends, rarefying the advantages of natural and synthetic excipients to achieve the desired drug release kinetics, mechanical strength, and stability (Mali et al., 2012). This combination of natural and synthetic excipients lends itself to applications in contemporary drug delivery systems, including hydrogels, nanoparticles, and matrix (Bhosale et al., 2014).

In conclusion, the swelling, gelling, matrix-forming, and biodegradable properties of *Hibiscus rosa-sinensis* mucilage provide a strong basis for its application in sustained release formulations. It functional versatility, together with compatibility with synthetic excipients, supports its potential as an innovative eco-friendly excipient for development of controlled drug delivery systems in the delivery of the antidiabetic drug metformin and Table 1. Functional characteristics of *Hibiscus rosa-sinensis* mucilage compared with other natural polymers. This table compares the key functional and physicochemical properties of *Hibiscus rosa-sinensis* mucilage with other natural polymers such as guar gum, xanthan gum, and sodium alginate. It highlights parameters like viscosity, swelling index, solubility, and binding capacity, emphasizing its excellent film-forming, bioadhesive, and biodegradable nature, making it a promising natural excipient for pharmaceutical formulations.

**TABLE 1:** Functional characteristics of *Hibiscus rosa-sinensis* mucilage compared with other natural polymers.

Property	*Hibiscus rosa-sinensis* mucilage	Guar gum	Xanthan gum	Pectin
Swelling Index and Water Retention	High swelling capacity; forms hydrated gel matrices suitable for controlled release	High swelling; often used in matrix tablets	Moderate; forms viscous solutions	Moderate; swells but less than Hibiscus and guar
Gelling and Thickening Capacity	Strong gelling and thickening; maintains viscosity over time	Good thickening; synergistic with other gums	Excellent viscosity modifier; stable across pH and temperature	Gel-forming, especially in presence of Ca^2+^ ions
Matrix-Forming Ability	Forms stable, robust matrices for sustained release tablets	Forms firm matrices but may require blending	Forms weaker matrices; often used with other excipients	Forms gel-like matrices but prone to erosion
Degradability and Protection	Biodegradable, non-toxic, highly biocompatible	Biodegradable, safe, but may cause GI discomfort in high amounts	Biodegradable, safe, widely GRAS-approved	Biodegradable, safe, widely used in food/pharma
Compatibility with Synthetic Polymers	Good compatibility; blends enhance sustained release	Good; often used with HPMC or PEO	Compatible with carbopol, HPMC	Compatible with synthetic polymers but less commonly used in blends
Unique Advantage	Combines swelling, gelling, and matrix stability in one polymer; eco-friendly and cost-effective	Excellent swelling but needs modification for strength	Superior viscosity stability under stress	Natural gelling, good for colon-targeting

The comparative analysis has demonstrated that *Hibiscus rosa-sinensis* mucilage provides a unique combination of high swelling index, high gelling capacity, and stable matrix formation and differentiation from other natural polymers. Guar gum will swell and provide high viscosity, and xanthan gum is effective for viscosity modification, however, on their own do not provide sufficient sustained release properties requiring mixtures with other products to provide optimal sustained release properties. Pectin can also be included in the formulation for its swelling and thickening properties.

Table 2 Summarizes numerical values associated with rheological properties, swelling behaviour, and drug-release kinetics of *Hibiscus rosa-sinensis* mucilage in various sustained-release formulations. The compiled data provide mechanistic evidence supporting its suitability as a natural polymer for controlled drug delivery applications.

**TABLE 2:** Quantitative physicochemical and release characteristics of *Hibiscus rosa-sinensis* mucilage-based sustained-release formulations reported in literature.

S. No	Type of study	Key numerical data/Properties	Outcome (release/Behavior)
1	Ketoprofen (matrix tablet with HRS mucilage) (Kaleemullah et al., 2017)	Dried mucilage powder: moisture content 14.70% ± 0.77%, weight-loss on drying 12.82% ± 0.58%; flow: angle of repose 25.37° ± 0.434°, bulk density 0.75 ± 0.014 g/cm^3^, tapped density 0.83 ± 0.017 g/cm^3^, Carr’s index 9.44% ± 0.178%, Hausner ratio 1.10 ± 0.002	With 40% mucilage polymer: sustained release up to 24 h; release kinetics followed non-Fickian (diffusion + erosion) mechanism
2	Gabapentin (sustained-release matrix tablet) (Ghumman et al., 2019)	Formulations F1–F5 with increasing mucilage content: % release at unspecified time points: 91.24%, 80.24%, 70.53%, 62.12%, and 49.83% respectively	Optimized high-mucilage batch (F5) showed ∼50% release → demonstrates strong release retardation by HRS mucilage; kinetic analysis (Korsmeyer–Peppas) gave release exponent n = 0.78 (non-Fickian)
3	Diclofenac sodium (matrix tablet) (Cetin and Sahin, 2016)	Dried mucilage: swelling ratio ≈9 in water, 10 in 0.1 N HCl, 9 in phosphate buffer pH 6.8; mass-loss on drying of mucilage ∼10.61%; powder flow: angle of repose ∼27.8°, “excellent” compressibility. Pharmaceutical Technology+1	Optimized batch (drug: mucilage 1:1.5 w/w) achieved sustained release ∼12 h; release kinetics best fit zero-order model; dissolution profiles in water vs. buffer nearly identical (similarity factor f_2_ ≈ 90.7), indicating pH-independent release
4	Ciprofloxacin hydrochloride (wet-granulation matrix tablets with HRS mucilage ± HPMC) (Ameer et al., 2025)	Swelling index of polymer mixture ranged 51%–211% over 10 h (depends on formulation). sciensage.info	Some formulations gave ∼65–67% cumulative drug release at 24 h, indicating prolonged release capability with HRS mucilage-based matrices
5	Domperidone (gastro-retentive floating tablet with HRS mucilage) (Srujan et al., 2018)	Post-compression parameters acceptable; release over 18 h: cumulative % release ranged from 81.37% to 98.62% depending on formulation	Optimized HRS-mucilage based floating tablets sustained release up to 18 h; kinetics fit zero-order/non-Fickian depending on batch — showing potential for controlled gastric release
6	HRS mucilage (as superdisintegrant in FDT) — (Amin et al., 2023)	Viscosity: 491.33 ± 119.44 cPs (2% w/v), 4520 ± 1224.42 cPs (4% w/v); particle size distribution: Dv(10) = 26.2 µm, Dv(50) = 157 μm, Dv(90) = 260 μm, Dv(100) = 380 μm; compressibility index 26.75% ± 1.79%	Though used as disintegrant (not sustained-release), these data illustrate rheological and powder properties — relevant for assessing polymer behaviour (hydration, viscosity, compressibility)

## 4 Metformin and the need for sustained release systems

### 4.1 Pharmacokinetic challenges

Metformin is a commonly used first-line oral antihyperglycemic agent for the treatment of type 2 diabetes mellitus. While metformin has helpful efficacy, it still poses significant pharmacokinetic constraints, limiting the potential for optimal therapeutic success (Jabbour and Ziring, 2011). Metformin has an approximate plasma half-life of 4–6 h and also exhibits incomplete gastrointestinal absorption, with bioavailability ranging from 50% to 60% based on the administered drug dosage. Consequently, metformin requires multiple daily dosing (2–3 times per day), potentially impacting adherence to therapy (Donnelly et al., 2009).

Besides potential pharmacokinetic issues, metformin therapy is often accompanied by gastrointestinal (GI) adverse effects: diarrhea, nausea, bloating, and abdominal pain. These GI adverse effects are likely due to the high local concentration of metformin, which is influenced by the rapid absorption and elimination of the drug (Abbasi et al., 2024). More specifically, the adverse effects of diarrhea and other GI tract adverse effects are common and often lead to intolerability requiring discontinuation or dose reductions of metformin and ultimately reduced long-term efficacy for optimal glycemic control. Accordingly, existing rapid release metformin formulations have significant limitations for patient tolerability, adherence to metformin therapy, and sustained therapeutic activity (Hu et al., 2006).

### 4.2 Benefits of sustained release metformin

To address these limitations, it is useful to formulate sustained release (SR) formulations of metformin. Sustained release (SR) systems change the release profile of the drug; SR profiles deliver the drug in a slow and sustained manner, resulting in stable plasma concentrations of the drug for a longer time (Jabbour and Ziring, 2011). This is beneficial as SR dosing can enable the ability to be dosed once daily per day which is important for convenience and adherence in patients with chronic illness such as diabetes (Donnelly et al., 2009).

In addition, SR metformin also improves glycemic control through the stabilization of therapeutic drug concentration and decreasing fluctuations of glucose levels in the blood which also decreases the risk of treatment failure (Tan et al., 2021).

In conclusion, sustained release metformin formulations not only address the pharmacokinetics limitations but also may have in improving the quality of life of patients and the long-term management of diabetes mellitus.

## 5 Prospects of hibiscus mucilage in metformin sustained release

### 5.1 Formulation approaches

*Hibiscus rosa-sinensis* mucilage has unique physicochemical characteristics that support high swellability, gel forming and biocompatibility, which could be utilized to formulate a natural polymer for sustained release formulations of metformin (Panda, 2025). These properties can be utilized for different formulation strategies to achieve different drug release kinetics (Amiri et al., 2021).

#### 5.1.1 Matrix tablets

The simplest and most commonly used sustained release formulations are matrix tablets. In these matrices, hibiscus mucilage can be incorporated into matrix tablets by direct compression and/or wet granulation to produce hydrophilic matrix tablets (Nayak et al., 2019).

#### 5.1.2 Hydrogels and beads

Hibiscus mucilage can be cross-linked or mixed with other natural/synthetic polymers to create hydrogels or polymeric beads. These three-dimensional networks can absorb water and swell, which encloses the drug and permits diffusion and matrix erosion, two mechanisms of controlled release (Yari et al., 2020). Hydrogel-based systems may also afford an advantage in the oral delivery of metformin, as they protect against premature dissolution and provide uniform release kinetics. Bead-based systems may also be utilized for site-specific delivery (Nayak and Hasnain, 2019).

#### 5.1.3 Nanoparticle-based formulations

Mucilage can also act as a stabilizer or matrix-former in the nanoparticle formulation process, maximizing the encapsulation efficiency of the drug, and regulating release kinetics at the nanoscale. Hibiscus mucilage formulations of nanoparticles may provide even further benefits, including increased surface area, improved dissolution, and possible mucoadhesion in the GI tract (Zolkepli et al., 2022). This can lead to a yet further reduction in dosing frequency, and may also improve the bioavailability of metformin due to the restriction to the small bowel of metformin absorption. Overall, *Hibiscus rosa-sinensis* mucilage is a natural, safe, and low-cost approach in these formulation strategies to develop systems with controlled release of metformin that may improve patient compliance, reduce the incidence of gastrointestinal side effects, and assist in optimizing glycemic control in type 2 diabetes (Upadhyay et al., 2022).

### 5.2 Mechanism of drug release

The controlled release of metformin from formulations with *Hibiscus rosa-sinensis* mucilage, will be based primarily upon the swelling and gel-forming properties of the mucilage (Anand et al., 2017). Upon contact with gastrointestinal fluids, the mucilage will swell in water via its hydrophilic polysaccharides, forming a gel-like layer around the dosage form. This gel-like layer can then act as a diffusion barrier to slow the release of metformin from the dosage form into the surrounding medium (Gopalakrishna Murthy et al., 2023).

In addition to diffusion, the mucilage matrix will also aid in the release of drug through its erosion. The matrix erodes slowly as the polymer continues to hydrate and degrade in physiological conditions, allowing the controlled release of the encapsulated drug. This mechanism combines the swelling-controlled diffusion with erosion, yielding a more reliable and extended drug release profile (Yahaya et al., 2023). Depending upon the formulation parameters such as the mucilage concentration, cross-linking and geometry of the tablets the drug release can follow either zero-order kinetics (constant release rate) or Higuchi kinetics (drug release is proportional to the square root of time). Given the biopharmaceutical profile of metformin, the utilization of both mechanisms is highly advantageous, as it enables the sustained regulation of its plasma concentration over an extended period, thereby enhancing therapeutic efficacy (Jani and Shah, 2008).

**FIGURE 3:**
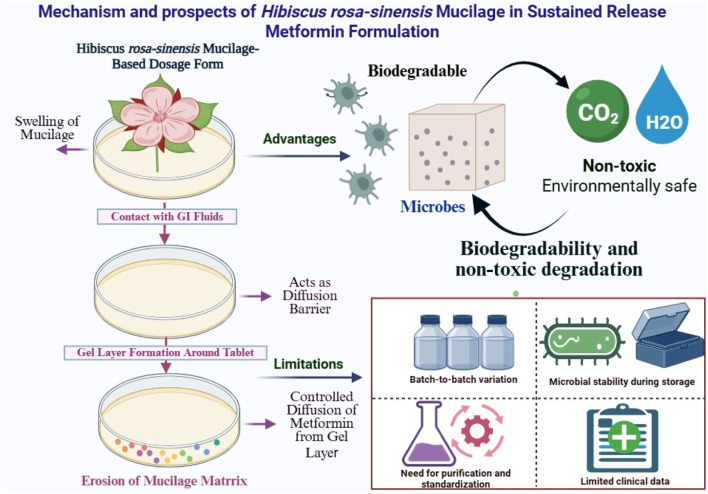
Schematic representation of the mechanism and prospects of *Hibiscus rosa-sinensis* mucilage in sustained-release metformin formulations.

The Figure 3, illustrates the role of *Hibiscus rosa-sinensis* mucilage in modulating the release kinetics of metformin through matrix formation, swelling, and gel barrier mechanisms. It also highlights its potential as a natural, biodegradable polymer offering controlled drug release, improved bioavailability, and enhanced patient compliance.

### 5.3 Advantages over synthetic polymers

The polymeric nature of natural polymer mucilage from *Hibiscus rosa-sinensis* offers a number of advantages compare to standard traditional synthetic excipients for sustained release formulations: Bio-resorbable and non-toxicity: The mucilage is naturally derived meaning it is biocompatible and will be metabolized and/or excreted without concerns for related risk from long period exposure to polymers from other sources and these are very cheaper (Maitz, 2015). Renewable and patient acceptance: Assuming the polymer will originate from a plant, the mucilage is renewable and acceptable wherever patients are looking for natural, plant based, or excipient free alternatives (Hacker and Mikos, 2011).

Product bioactivity: Beyond the mucilage’s functional use as an excipient, it has been reported, the mucilage itself contains antioxidant and anti-inflammatory activity that may further benefit the patient. These bioactivities may help modulate oxidative stress and inflammation for patients with diabetes, potentially adding therapeutic value to the metformin formulation (Gunatillake et al., 2006).

Together, these benefits demonstrate the applicability of Hibiscus mucilage as both a functional excipient, and a value-added ingredient that could potentially enhance overall health outcomes in improved diabetes care (Satchanska et al., 2024).

### 5.4 Limitations and challenges

Various obstacles exist to potential applications of *Hibiscus rosa-sinensis* mucilage as a sustained release formulation. These include.

#### 5.4.1 Variability between batches

When using natural polymer(s), variability occurs due to plant source, growing conditions and time of year causing variation in composition and functional characteristics that may result in variance in drug release (Ameena et al., 2010). Standardizing cultivation methods, as well as harvesting and extraction methods along with detailed physicochemical analysis will assist with minimization of this.

#### 5.4.2 Microbial stability

Due to high polysaccharide content the mucilage has an enhanced risk of bacterial contamination during storage; thus affecting the safety of the formulation. Additionally, the use of natural preservatives (sodium benzoate), The sterilization methods (autoclaving, gamma irradiation), as well as spray drying may be used to improve microbial stability without changing the functional characteristics of the mucilage (Taşdemir et al., 2020).

#### 5.4.3 Requirements for purification and standardization

The requirements for purity, consistency, and characterization of mucilage will add complexity to the formulation; thus, it must be extensively purified and standardized in order to provide consistent drug release Methods available for purifying mucilage include ethanol precipitation, ultrafiltration, or dialysis. Analytical techniques (e.g., viscosity measurements, swelling index, and molecular weight) can be used in conjunction with the use of purification methods to achieve batch uniformity and predictable performance of mucilage (Li et al., 2024).

#### 5.4.4 Lack of clinical and regulatory information

In comparison to synthetic polymers (e.g., HPMC and carbopol), an absence of clinical studies and regulatory agency guidelines has hindered the use of Hibiscus mucilage as a suitable pharmaceutical grade excipient. Conducting systematic preclinical and clinical evaluations, and adhering to appropriate regulatory standards (e.g., GRAS and FDA/EMA excipient guidelines), are paramount in supporting the safe use of Hibiscus mucilage as an excipient in pharmaceutical formulation (Jejurkar and Chavan, 2023).

Through the employment of the strategies suggested above, *Hibiscus rosa-sinensis* mucilage may overcome the present limitations to its reliability and clinical applicability as a sustained-release formulation (Amtaghri et al., 2024).

## 6 Future perspectives

The growing interest in the utilization of natural polymers for sustained release of pharmaceutical agents suggests that *Hibiscus rosa-sinensis* mucilage could serve as a drug-excipient in the regulation of diabetes (Anderson, 2006). Future study and development can take various opportunistic paths to achieve the pharmaceutical benefit of Hibiscus mucilage.

### 6.1 Polymer blends

One avenue to begin would be known development of polymers mixtures, whereby Hibiscus mucilage could be mixed with well-characterized synthetic polymers such as hydroxypropyl methylcellulose (HPMC), polyvinylpyrrolidone (PVP), or carbopol to produce an optimal drug release profile, mechanical properties, and stability of sustained release dosage forms (Selvan and Ramesh, 2024). With careful adjustment of the ratio and properties of each synthetic and natural polymer, formulations may achieve controlled and predictable drug release, eliminating some variability inherent in using natural polymers alone (Sahu et al., 2024).

### 6.2 Novel dosage forms

In addition to matrix tablets, Hibiscus mucilage use could be extended to a variety of advanced drug delivery systems, including microspheres, nanoparticles and a variety of mucoadhesive films. Microspheres and nanoparticles allow highly controlled drug release, enhanced absorption, and drug targeting to specific regions of the GI tract (Barus et al., 2025). Mucoadhesive films allow potentially prolonged residence time of a drug in the oral mucosa or the intestinal mucosa, which is especially useful for drugs like metformin, which have small absorption windows. The development of dosage forms using Hibiscus mucilage in these various designs may create an opportunity to extend individualized and patient-friendly diabetes control (Yahaya et al., 2023).

### 6.3 Pharmacological synergy

Additionally, to possible excipient properties, Hibiscus mucilage may elicit pharmacological properties. Evidence presented in the literature suggests that extracts of Hibiscus have anti-diabetic, antioxidant, and anti-inflammatory properties (Kapoor et al., 2021). Future formulations in developments may utilize the potential synergistic activity of the mucilage in which mucilage would act to control drug release and simultaneously to facilitate glycemia control and reduce oxidative stress activity while increasing the overall efficacy of metformin regimens (Abate and Belay, 2022).

### 6.4 Clinical translation

Despite a large body of preclinical evidence for Hibiscus mucilage, clinical applications remain unrealized. A systematic and comprehensive program of preclinical and clinical research will be necessary to define reliability, efficacy, and pharmacokinetic potential of mucilage as sustained released formulations (Yasmin et al., 2023). Clinical studies to examine certain patient outcomes, tolerate, and longer-term compliance will be of necessity to parametrically introduce a commercial pharmaceutical product for the end user. In addition, regulatory standards and evidence based standardized protocols will be required for extraction of the mucilage to prepare it to become a part of diabetes therapy (Nogueira Silva Lima et al., 2022).

### 6.5 Future possibilities of regulation and standardization

Natural Excipients such as *Hibiscus rosa-sinensis* mucilage have the potential for safe and sustainable pharmaceutical delivery but must adhere to regulatory guidelines (e.g., FDA/EMA) for safety, quality, and reproducibility in order to gain greater use. To meet this challenge, it will be necessary to develop standardized methods for extracting, purifying, and characterizing natural excipients as well as develop pharmacopoeia monographs to ensure that the functional properties of each natural excipient are consistent from one batch to the next and that they may be used to create commercial pharmaceutical preparations (Kavoosi et al., 2025; Amin et al., 2023).

**FIGURE 4:**
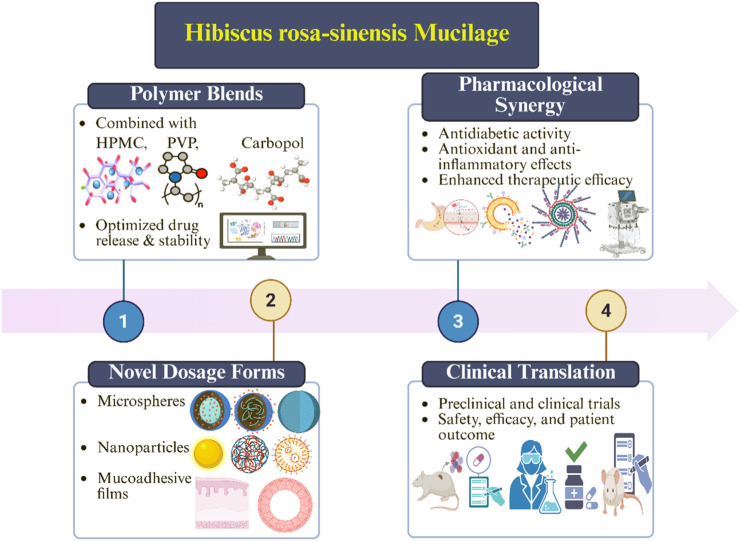
Schematic representation illustrating the future perspectives of *Hibiscus rosa-sinensis* mucilage in the sustained release of Metformin.

In conclusion, the potential of *Hibiscus rosa-sinensis* mucilage to be formulated for drug delivery will largely depend on the methods of formulating mucilage with contemporary formulation techniques, the delivery of the mucilage in novel dosage forms, as well as comprehensive clinical studies for human subjects (Saeed et al., 2025; Nguyen et al., 2025). The future of *Hibiscus rosa-sinensis* mucilage is to utilize the unique functional capabilities of natural polymers through their effects and properties that can provide sustained release of a therapy that is safe, effective, and patients friendly for managing diabetes (Amin et al., 2023; Amin et al., 2022). The detail future prospective were depicted in Figure 4.

## 7 Conclusion

The exploration of natural polymers in the role of pharmaceutical excipient has been stimulated based on stability, biodegradable products, and economical. Of the natural polymers evaluated, *Hibiscus rosa-sinensis* mucilage could serve as an option for sustained release. Due to hibiscus’s high swelling capacity, gel-forming capabilities, and matrix stabilizing properties, it is well suited for extended release of metformin which has limited therapeutic value due to short half-life, often not fully absorbed, and is often associated with gastrointestinal effects. Hibiscus mucilage could provide a depletion of hydrated systems and modulate drug diffusion and erosion, providing stable plasma drug concentrations. In addition to their conventional synthetic polymer counterparts, Hibiscus mucilage has additional advantages, including renewable sourcing, patient acceptability, and potential pharmacological action due to antioxidant and antidiabetic effects. Formulation strategies such as matrix tablets, hydrogels, beads, and nanoparticles may be used to take advantage of these functional attributes, and polymer blends with synthetic excipients may provide additional control over release kinetics and mechanical strength.

Despite the potential of Hibiscus mucilage for pharmaceutical development, challenges remain, including batch-to-batch variability, stability and growth of the mucilage’s microflora, and the need to purify and standardize the mucilage for clinical use, all of which serve to underscore the importance of thorough characterization and quality assurance. Importantly, the current lack of a more robust dataset from preclinical and human clinical research studies suggests there are still outstanding questions regarding the safety, efficacy, and overall outcomes within the patient population when applying Hibiscus mucilage as a sustained release delivery system.

In summary, *Hibiscus rosa-sinensis* mucilage has the capacity to be a natural, multifaceted polymer in the development of sustained release systems for metformin. If developed as a sustained release metformin delivery system, the addition of Hibiscus mucilage to pharmaceutical technology combined with a comprehensive evaluation of clinical data evidences a potential shift for safe, effective, and patient-friendly care for diabetes, while providing potentially better glycemic control and improved quality of life for patients with type 2 diabetes.
